# Consumer Flow Experience of Senior Citizens in Using Social Media for Online Shopping

**DOI:** 10.3389/fpsyg.2021.732104

**Published:** 2021-09-15

**Authors:** Ying Xu, Yixuan Wang, Asif Khan, Ranlin Zhao

**Affiliations:** ^1^School of Management, Jilin University, Changchun, China; ^2^Department of Business Administration, School of Management, Jilin University, Changchun, China; ^3^Department of Marketing and Distribution Management, College of Management, National Kaohsiung University of Science and Technology, Kaohsiung, Taiwan; ^4^School of Management, Universiti Sains Malaysia, Penang, Malaysia

**Keywords:** senior market, flow experience, antecedents of flow, technology acceptance model, social media purchase intention, PLS SEM

## Abstract

The senior market signifies an enormous, rapidly expanding segment, and this research aimed to investigate this segment by proposing a theoretical model incorporating the antecedents of consumer flow experience, flow theory, and technology acceptance model (TAM) devised for determining social media purchase intention. This study focuses on the senior citizens engaged in shopping using social media located in Pakistan. A total of 300 senior citizens were selected. An online survey was conducted with the help of a marketing research agency located in Pakistan. The data were analyzed using the partial least squares (PLS) method. According to the results, the antecedents, such as feedback, enjoyment, and time distortion were found to be in a positive relationship with flow experience, however, the concentration did not have a significant effect on the flow. Furthermore, the flow was found to be in a significant relationship with social media purchase intention and TAM. Finally, TAM was also found to be in a positive significant relationship with social media purchase intention. This research contributes to the constantly expanding volume of the utilization of social media by the senior market segment population for buying and producing the highly valuable knowledge for manufacturers, wholesalers, vendors, and a huge number of senior customers.

## Introduction

Seniors continue to signify an essential and rapidly increasing percentage of the population in numerous developed economies that can accept a more influential place in shopping using social networking sites given their buying power and wealth (Kim et al., 2016b). According to the prior studies, this research identifies individuals that are aged 50 years and older as senior citizens (Anderson and Langmeyer, 1982; Kim et al., 2016a,b). A constant and advanced sequence of useful understandings of how social networking sites (SNSs) will be able to utilize to promote products and services, for the success of businesses via advertising, sales development, and connections with consumers has initiated an increase in SNSs (Yadav et al., 2015). The increasing significance of technologies in advertising has created a massive curiosity in the educational and industrial world in cultivating desirable encounters to present to online customers. To create these experiences, it is essential to analyze states related to flow. This state is an ideal psychological capability, linked with enjoyment in conducting a task, and forecasting online customer behavior. In electronic businesses, the flow and the optimistic attitudes it produces, boost intent to purchase, endorse online sites, and maintain usage. Consequently, in e-commerce companies, flow state is vital and would be studied to recognize the customer behavior and to enhance the interactions between customers and the company in virtual settings. Furthermore, online situations have features that enable flow, for instance, their level of communication. More precisely, the e-commerce environment allows customers to focus and lose the trail of time in the course of their collaborations, letting them appreciate the flow activities (Barta et al., 2021). Corresponding to the earlier research, flow experience produces a constructive emotional state for senior citizens that can demonstrate pleasure, fulfillment, and profound life, and allows adaptable and innovative thinking motivating to a sense of emotional strength. Senior citizens usually experience flow once they get involved in the behaviors possessing suitable degrees of skills and abilities. The relaxation pursuits of senior citizens are significantly associated with their experiences produced whenever they are in a state of flow. Furthermore, retirement is adversely associated with their experience of flow (Heo et al., 2010). Senior citizens with greater degrees of flow are linked in conjunction with a greater psychological state that is related to the constructive influences, for instance, being livelier, more excited, having positive attitudes, and a feeling of life fulfillment (Kim et al., 2017). Based on the existing studies, this research anticipates that use of SNSs and flow experiences are significantly correlated with senior citizens. Thus, this study utilizes the antecedents of measuring consumer flow experience from Barta et al. (2021) study as feedback, concentration, enjoyment, and time distortion experienced by the senior citizens while using social media for shopping.

The outstanding growth of SNSs has converted an online social communication platform to a virtual social electronic commerce and offered chances for several electronic retailers to connect with online customers and perform business activities with them. This swift development of social networking sites has significantly enhanced the number of social networking sites customers on sites, namely, Facebook, Twitter, and Instagram. SNSs present online customers with a variety of convenient buying opportunities for products and useful methods of cooperating with more customers. SNSs must turn out to be a vital platform for merchandise search among the customers. Moreover, as SNSs have developed globally, marketing initiatives are considered more essential. Therefore, individuals have been allowed by SNSs to alter their buying activities and the means that they use to communicate with businesses (Hyun et al., 2021). Earlier scholars have built numerous theoretical frameworks integrating a variety of factors to display the tendency of customer activities in the societal marketplace that suggest the concept change in the path of identifying recent activities of SNSs customers and their social purchasing events. Conveyed the significance of customers of SNSs and their online social spending behaviors, numerous studies have a scarce correlation between flow and perceived ease of use (PEOU) and perceived usefulness (PU) of SNSs for purchasing. Hence, it can be inferred that buying intention on SNSs centered on flow theory and technology acceptance model has obtained less awareness in the perspective of social business. Additionally, analyzing the impact of the correlation between flow experiences of customers and technological attitudes on the outcome of customers to shop using SNSs is important as numerous previous research have observed the part of the flow in traditional electronic commerce (Mahnke et al., 2015; Su et al., 2016). According to Yusuf and Busalim (2018), distinctions between traditional e-commerce and social commerce exist. This indicates that although there is growing attention in researching electronic commerce, social commerce deserves its theory creation to distinguish it from electronic commerce theories.

The existing research considers customers of SNSs may display a distinct type of buying flow experience in SNSs comparative to conventional websites. The necessary constructs, for instance, PU and PEOU of technology, develop views of customers toward innovative technology. The current research identifies PEOU as an idea that SNS usage needs less intense work, and PU implies the idea that SNS usage is under the associated shopping requirements. The recognition and use intent of innovative technological products, comprising online buying and social networking site use, are mostly impacted by these belief components in the technology acceptance model (TAM) (Chen Y.-M. et al., 2018). SNSs have turned out to be an outstanding platform for buying. Consequently, examining the antecedents of users of SNS flow and possible consequences can be further pertinent to the existing and upcoming trends in research. Nevertheless, the number of research studying flow experience and the use of SNSs for shopping are yet deficient. Though earlier studies show that flow state of users is a crucial component in online buying (Su et al., 2016), buying via SNSs involves a unique theoretical viewpoint. Since, studies focusing on flow experience social media shopping are significantly less, incorporating the theory of flow with TAM and analyzing flow on social shopping intention can develop a frame of understanding of why individuals choose to utilize SNSs to shop (Hyun et al., 2021).

This research covers at least four research gaps. First, it covers the antecedents responsible for generating or affecting the flow experience of senior online shoppers while using social media. Second, it analyzes the correlation between the flow experience and SNS buying intention of senior shoppers. Third, it explores the correlation between flow experience and the TAM model. Finally, it analyzes the relationship between the TAM model and social media purchase intention. The current study can benefit researchers to increase a greater understanding of social buying behavior of customers and support experts to improve their existing SNSs marketing plans and articulate SNS tactical options for upcoming operations by exploring the novel mechanism of the theoretical framework comprising the antecedents of flow, flow theory, and TAM.

## Theoretical Background

### Flow Experience

Flow experience indicates an emotional status of a person when completely involved in an action or engaged in an experience (Csikszentmihalyi and Csikzentmihaly, 1990). Flow implies an enhanced status of focus, strength, and concentration when participating in a particular action or even in a variety of day-to-day events, for instance, watching a movie or performing sports activities (Ettis, 2017). Csikszentmihalyi and Csikzentmihaly (1990) describes flow as a condition in which an individual is so entirely absorbed in what they perform with an enjoyable feeling that additional nearby stimuli tend to be ignored. Especially, individuals are absorbed in an activity that can simply concentrate on a particular purpose while disregarding the presence of other objects, and then sense enjoyment and satisfaction. Flow experience is a transitory and subjective psychological status deemed as the optimal experience situation for customers. Moreover, flow experience is the top indicator to indicate the loyalty of customers (Ding and Hung, 2021). Gaining flow experience is the key motivation for people to join leisure events (Cheng and Lu, 2015). Numerous areas have utilized flow theory widely, particularly, in social media usage conduct (Lin et al., 2020), online shopping (Gao and Bai, 2014), mobile shopping (Chen Y.-M. et al., 2018), and information technology (Teng et al., 2012). Novak et al. (2000) refer to the web-based flow position as a cognitive situation responsible for smooth replies and the level of interaction in online settings, internal enjoyment experience of an individual, failure of their self-reinforcement, and self-awareness. Individuals in the situation of having a flow experience face nice and enjoyable encounters and have a high-level of control on behavior while engaging in an activity. Therefore, individuals in a state of flow are engaged in events, attaining a greater degree of attention and enjoyment, suggesting the seamless activities taking place while engaging in tasks. Moreover, as the performer appears to filter out unenjoyable emotional encounters, the act is performed without disruption. It allows the performer a state of power over the act that includes implementing a specific activity involving both abilities and tasks, either practical or uncomplicated, letting the users of SNSs understand flow (Hyun et al., 2021). This experience of flow is a combination of emotional conditions that users can become entirely engaged within a stimulus without observing prospective hazards (Wang et al., 2017) and adverse events (Lin et al., 2020).

### Technology Acceptance Model

The TAM proposed by Davis (1993) is built facets of social psychology and on innovation diffusion theory and offers a helpful instrument for studying the interaction and acceptance of novelties and concepts. In investigating the motives of users for recognizing (or refusing) the latest scientific innovations, the technology acceptance model utilizes two methods, such as the PU and PEOU, to calculate the ultimate choice of users. The framework has been generally utilized to analyze the customer reaction in several research fields, involving information technologies associated with SNSs (Do et al., 2020). The web-based application use and its recognition have been commonly explored using the TAM, which combines two major concepts, for instance, PEOU (Besbes et al., 2016). The theory of reasoned action and information systems indicate that PU and PEOU are crucial analysts of implementation intention for social media and website services (Hyun et al., 2021). This suggests that the two important TAM concepts have a substantial effect on SNS acceptance intent in several SNS shopping programs (Chen and Barnes, 2007). Consequently, it is acceptable to implement flow theory and assess its influence on the TAM and the entire procedure of customer social buying intention. This existing research recommends that the construct of flow is essential for identifying the social buying conduct of a customer and its impact on the TAM. As flow is a condition of fundamental pleasure that raises the readiness of an individual to execute a specific assignment because of its enjoyment and satisfaction, it describes the experiences of an individual of the task, which make it appear valuable and effortless. Intrinsically, the connection between the condition of flow of a person and the communication with several social networking buying sites recommends that it may be recognized as pleasant and enjoyable, leading to the deepest commitment of shopping behaviors (Hyun et al., 2021).

## Hypothesis Development

**Figure 1:**
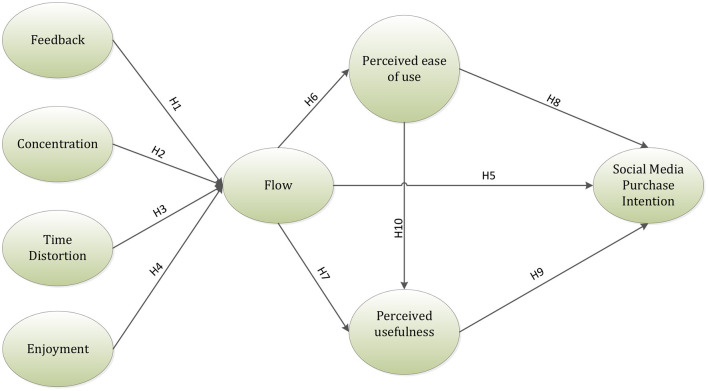
Research framework.

Based on the analysis of the specific literature on theory of flow and information systems, a research framework is proposed (Figure 1). The framework recommends that four antecedents affect customers to have flow encounters. Furthermore, it depicts the impact of flow on TAM and in turn depicts the impact of TAM on social media purchase intention. Flow experience is described by a great series of activities, in which a person is focused on a particular task and appreciates the action which the individual is performing, leads to customer gratification. This optimistic understanding affects the earlier held beliefs of customers to be, however, if met, makes the individual gratified. The satisfaction produced makes the customer intend to replicate the experience and additionally reinforces the great encounters an individual has had browsing on the internet.

### Antecedents of Flow

#### Feedback and Flow

Feedback refers to the mutual knowledge flow method (Voorveld et al., 2011). In the web perspective, it describes the exchange of information between users and the websites (Huang, 2003). Once the user believes in command of the human-technology collaboration, the feedback method is improved. It creates experiences of competency and self-assurance that improve independence and, as a result, the sense that one possesses sufficient abilities to tackle the task. The feedback that has been explored in numerous research fields, is found to enable the creation of flow states and is demonstrated to be a key element in the achievement of online marketing (Barta et al., 2021). Once people obtain prompt and transparent feedback on the actions they are performing, they can decide whether their competence levels are sufficient for the activities they are working on. In contrast, when the users are oblivious of the outcomes of the activities performed by them during navigation, the flow experience might get blocked. The users in this scenario are found to be unaware of their success related to their tasks, and, hence, if their capabilities are sufficient, this situation might lead to a condition of anxiety and worry for the users (Massimini, 1988). Therefore, the following hypothesis can be suggested:

H1. Feedback has a positive relationship with the flow experience.

#### Concentration and Flow

Concentration is described as the focus given to a restricted stimulus area. Concentration is found to be one of the highly established components in the analysis of flow; according to the findings, individuals ought to be concentrating on their situations (comprising the stimuli) to engage in flow experience (Barta et al., 2021). In describing the issues that impact the communications between individuals and technology, Csikszentmihalyi and Csikzentmihaly (1990) indicated that the attention paid by the individuals must be restricted to, or concentrated on, the limited stimuli offered by the technology. Consequently, in the web environment, users should emphasize all their focus on the cause of the stimuli, which in this case is the screen, to continuously engaged in experiencing flow, and lose awareness of all the additional aspects irrelevant to their actions (Barta et al., 2021). Some research related to web-based customer experiences have demonstrated that higher concentration is related to higher flow experience (Novak et al., 2000). In SNSs, the users are found to be usually encountering promotion or product suggestions that are based on the purchasing behavior of other users possessing somewhat similar traits to them. These stimuli might cause a distraction to the users, while they are engaged in performing their actions during the purchase procedure, especially in people with minimal levels of concentration. Nevertheless, people that are extremely concentrated on the actions they perform are found to be paying minimal interest to these stimuli, hence achieving a state of flow. Therefore, concentration is an essential construct in this framework, and the following hypothesis can be indicated:

H2. Concentration has a positive relationship with flow experience.

#### Time Distortion and Flow

Time distortion is described as a state where the individuals are observed to lose the perception of time and misunderstands the period of their actions (Chen et al., 2000). Time distortion is a fluctuating prerequisite for boosting the flow state of customers. Numerous theoretical frameworks of flow comprising computer-facilitated interface have indicated time distortion as an antecedent of flow (Hoffman and Novak, 1996; Novak et al., 2000). In web-based scenarios, the engagement of users with technology is found to stimulate the feeling of being carried away through space and time that enables the advent of flow experiences (Barta et al., 2021). Consequently, it can be hypothesized that:

H3. Time distortion has a positive relationship with flow experience.

#### Enjoyment and Flow

The definition of enjoyment as per the interactions between humans and technology is the level to which the user perceives the utilization of the system as a pleasurable experience, putting away any additional effects of its usage (Davis et al., 1992). Moreover, in the online perspective, enjoyment is defined as the feeling of the users while browsing the web, keeping in mind their area of interest, the content accessed by them, the medium used by them, and their level of satisfaction (Barta et al., 2021). The individuals using web-based activities find themselves engaged and immersed while performing those activities because of the fun and pleasurable environment. Consequently, it is found that the enjoyment experienced during online shopping activities is significantly related to flow experience (Kim et al., 2013). Numerous customer-behavior research targeting e-commerce indicated, as per the theory of flow that there will be an increase in flow whenever the observed hedonic value is high (Senecal et al., 2002). Hence, it can be hypothesized that enjoyment in online shopping will generate flow. Therefore, the following can be postulated:

H4. Enjoyment has a positive relationship with the flow experience.

### Flow and Social Media Purchase Intention

The relationship between flow and social media purchase intention can be built on the prior literature suggesting a web-based environment as a cause of flow, therefore it can be indicated that flow is an essential framework to explain user behavior in an online context (Hyun et al., 2021). According to the findings of Lim (2014), online flow experience is found to be in a significant relationship with the purchase intention and online shopping experience of the customers. Furthermore, Gao and Bai (2014) also indicated flow to be responsible for the significant boost in buying products related to travel and customer satisfaction in an online context. Flow indirectly affects the loyalty of users via the impacts of trust and brand equity associated with the owners of websites (Bilgihan et al., 2015). Additionally, in an online environment purchase intention of customers is significantly impacted by satisfaction, perceived command, and integration (Ozkara et al., 2017). Most of the prior literature suggests a significant relationship between flow and shopping on traditional e-commerce websites. However, research analyzing social media purchase behavior has also found a significant relationship between flow and social commerce purchase (Rahman et al., 2020). Therefore, the following can be hypothesized:

H5. Flow experience has a positive relationship with social media purchase intention.

### Flow and TAM

#### Flow and PEOU

As complete experiences, flow explains the theory of cognitive absorption (Saadé and Bahli, 2005) that signifies a fundamental motive (Kristof-Brown et al., 2005) of a specific action responsible to produce satisfaction and pleasure. This current study illustrates this theory as it emphasizes the vital aspect of comprehending the communication between technology and humans to hypothesize its connection with usefulness and ease of use view of SNS and purchase intention. The studies that incorporate the theory of flow with the TAM assert that individuals misperceive their time and appear to complete a task faster whenever they are in a condition of absorption that in turn enhances flow (Hyun et al., 2021). They also possess a lesser cognitive responsibility, causing the development of the ease of use perception, and thus, they can further enjoy the events (Saadé and Bahli, 2005). The current research examines this association, it is hypothesized that users of SNS would understand that social media shopping is simpler to utilize as they would consider that the purchase behavior is performed easily with no cognitive responsibility, utilizing less effort and time. This occurs as users of social media move into a state of flow in which their collaborations with SNSs are discovered to be so enjoyable and entertaining that further ideas appear to be ignored (Csikszentmihalyi and Csikzentmihaly, 1990). Therefore, the following hypothesis is postulated:

H6. Flow experience has a positive relationship with PEOU of social media shopping.

#### Flow and PU

Earlier research (Hyun et al., 2021) has tried to combine the theory of flow with the underlying TAM. They indicate that the degree of cognitive dissonance related to the implementation of technological activities is decreased when individuals feel the flow as they consider that using time on a specific job should be valuable (Agarwal and Karahanna, 2000). As indicated in the theory of self-perception by Bem (1972), individuals manage to justify their activities and attempts to lessen cognitive dissonance, such as mindsets, viewpoints, or actions. During the state of cognitive absorption, individuals experience satisfaction and enjoyment from engaging with technological activities, their conflicts are lessened whenever they are found to be in a pleasant and enjoyable state (Hyun et al., 2021). According to the concepts stated above, it is assumed that whenever buyers are essentially driven, they would recognize buying on SNSs as valuable. Thus, the following hypothesis is proposed:

H7. Flow experience has a positive relationship with the PU of social media shopping.

### TAM and Social Media Purchase Intention

#### PEOU and Social Media Purchase Intention

The capability to utilize an innovative approach easily signifies the idea of PEOU of a specific method. In this research, PEOU of social media sites for purchasing is theorized as easy to use, improving the pleasure and accessibility of social networking sites whenever buyers utilize the features included in social media shopping, for instance, diversity of products, simple payment, and purchasing procedures. Therefore, it results in a higher result of social media purchase intention (Hyun et al., 2021). According to a study by Yahia et al. (2018), it was found that intention was in a significant relationship with PEOU. Moreover, Hansen et al. (2018) demonstrated a significant association with the usage intention of social media for shopping. Consequently, this research suggests that PEOU is vital whenever examining the intentions of customers to engage in social media shopping, and hence it is one of the most important elements that can describe social media shopping behavior. Consequently, the following hypothesis is suggested:

H8. Perceived ease of use has a positive relationship with social media purchase intention.

#### PU and Social Media Purchase Intention

Davis (1993) describes PU as the attitude of an individual that their work performance might be improved by utilizing a specific method. It additionally implies the probability to raise productivity and efficacy in the entire buying procedure (Hyun et al., 2021). PU substantially affects conduct of SNSs via the use of attitude construct. Customers think that SNSs are beneficial for collecting and identifying product knowledge (Chen Y.-M. et al., 2018). PU substantially affects the intention to explore information (Gibreel et al., 2018). According to the study of Osatuyi et al. (2020), social media buying intention is significantly impacted by PU. These findings indicate that customers determine whether to utilize or reuse a specific social media to implement the buying process built on its usefulness. Hence, the following hypothesis can be suggested:

H9. Perceived usefulness has a positive relationship with social media purchase intention.

### Perceived Ease of Use and Perceived Usefulness

Perceived ease of use is the extent to which an individual considers that utilizing specific methods would be effortless (Taufik and Hanafiah, 2019). Moreover, Legris et al. (2003) in their research, reveal the major relationship between PEOU and PU. PEOU is found to be in a significant correlation between the PU and customer satisfaction in the online mobile web-based context (Amin et al., 2014). In accordance with the previous research, Chen Y.-M. et al. (2018) demonstrate that PEOU is a powerful determining factor of PU in the implementation of technical products. Additionally, PU, PEOU, and perceived behavioral control demonstrate a substantial impact on the social media usage intention (Shin and Perdue, 2019).

H10. Perceived usefulness has a positive relationship with PEOU.

## Methodology

### Sample and Procedure

This study focused on the social media purchase intentions of senior citizens. A total of 300 senior citizens were selected based on the convenience sampling method. Individuals of 50 years of age and over were selected for this study (Anderson and Langmeyer, 1982; Kim et al., 2016a,b). The selected senior citizen samples were users of Facebook, which is a popular and frequently used SNS in the country. An online survey was conducted with the help of a marketing research agency located in Pakistan. Rather than asking the respondents simply whether they agreed to an opinion statement, Likert scale items asked how strongly they agreed or disagreed with it, usually on a 7-point scale from 1 (= strongly disagree) to 7 (= strongly agree), with 4 being a neutral feeling or category.

The antecedents of flow experience were measured by items suggested by Barta et al. (2021), while the items to measure flow, social media purchase intention, PU, and ease of use were adopted and modified from Hyun et al. (2021) research study. The hypothesis of this study was tested using a partial least squares-structural equation model (PLS-SEM).

## Data Analysis

The evaluation and measurement of the partial least squares (PLS) were done in two phases. The reliability and validity of the framework were analyzed in the first phase, whereas the path coefficients were analyzed in the second phase of the data analysis. The reliability and validity of the constructs and their relationships were tested during the two phases (Anderson and Gerbing, 1988; Hulland, 1999). PLS is known as one of the best tools to empirically examine the relationships between the variables and manage the measurement constructs at the same time (Petter et al., 2007). Additionally, PLS is ideal in handling the constructs with irregular distribution because of the easy-to-use parameters of PLS for measuring the randomness and normality of constructs. PLS possesses the advantages of measuring dynamic prediction frameworks (Chin and Newsted, 1999). Hence, PLS was found to be more suitable in measuring and analyzing the construct relationships for this research study.

### Outer Model and Validation

The internal consistency and reliability of each construct were analyzed in the measurement of the external model. It also included the discriminant and convergent validity of each item. The reliability of each item was tested with an associated loading. The threshold factor loading value of 0.6 was used to determine the reliability of each item (Hair et al., 2010). Table 1 shows the composite reliability (CR) of each construct. The CR was found to be higher than the threshold CR value of 0.7 for each construct in this study, hence, indicating that each construct was internally significant (Chin, 1998).

**Table 1:** Convergent validity.

**Construct**	**Item code**	**Factor loading**	**Cronbach's alpha**	**Composite reliability**	**Average Variance Extracted (AVE)**
Concentration	CON1	0.758	0.874	0.9	0.692
	CON2	0.783			
	CON3	0.905			
	CON4	0.874			
Enjoyment	ENJ1	0.826	0.872	0.912	0.723
	ENJ2	0.875			
	ENJ3	0.846			
	ENJ4	0.852			
Feedback	FEED1	0.872	0.929	0.949	0.824
	FEED2	0.915			
	FEED3	0.923			
	FEED4	0.919			
Time distortion	TIME1	0.852	0.835	0.9	0.75
	TIME2	0.877			
	TIME3	0.87			
Flow	FLOW1	0.911	0.87	0.92	0.794
	FLOW2	0.908			
	FLOW3	0.852			
Perceived ease of use	PEOU1	0.889	0.885	0.929	0.813
	PEOU2	0.926			
	PEOU3	0.89			
Perceived usefulness	PU1	0.874	0.916	0.941	0.798
	PU2	0.931			
	PU3	0.884			
	PU4	0.885			
Social media purchase intention	SMPUR1	0.909	0.87	0.92	0.793
	SMPUR2	0.892			
	SMPUR3	0.871			

The average variance (AVE) calculated for each construct was also considered to measure the convergent validity. The values were analyzed with the threshold AVE value of 0.5. It was found that all the constructs possessed an AVE value higher than the threshold value of 0.5, hence, suggesting a good convergent validity (Fornell and Larcker, 1981). Table 1 uncovers that the AVEs for the constructs measured in this evaluation of study are between 0.692 and 0.824, implying a significant convergence.

Discriminant validity was used to measure the differences between the various constructs and research items. Table 2 suggests decent discriminant validity for all the indicators measuring the constructs. The factor loading value of every indicator measuring a specific construct exceeds the factor loading value of all the other constructs in the latent structure (Hair et al., 2016).

**Table 2:** Standardized factor loadings and cross-loadings of the outer model.

	**Concentration**	**Enjoyment**	**Feedback**	**Time distortion**	**Flow**	**Perceived ease of use**	**Perceived usefulness**	**Social media purchase intention**
CON1	0.758	0.367	0.428	0.496	0.209	0.378	0.300	0.434
CON2	0.783	0.368	0.432	0.458	0.239	0.390	0.329	0.436
CON3	0.905	0.505	0.538	0.597	0.411	0.453	0.517	0.541
CON4	0.874	0.663	0.638	0.710	0.646	0.640	0.688	0.695
ENJ1	0.535	0.826	0.436	0.655	0.553	0.521	0.516	0.566
ENJ2	0.531	0.875	0.514	0.669	0.578	0.461	0.544	0.597
ENJ3	0.503	0.846	0.490	0.573	0.550	0.456	0.512	0.541
ENJ4	0.555	0.852	0.533	0.641	0.529	0.462	0.531	0.571
FEED1	0.609	0.536	0.872	0.586	0.614	0.532	0.657	0.633
FEED2	0.599	0.514	0.915	0.575	0.589	0.432	0.668	0.589
FEED3	0.536	0.502	0.923	0.574	0.631	0.455	0.730	0.605
FEED4	0.598	0.551	0.919	0.633	0.648	0.561	0.716	0.662
TIME1	0.623	0.720	0.522	0.852	0.657	0.562	0.604	0.678
TIME2	0.627	0.613	0.579	0.877	0.505	0.505	0.531	0.665
TIME3	0.605	0.586	0.604	0.870	0.531	0.452	0.591	0.681
FLOW1	0.425	0.601	0.543	0.547	0.911	0.510	0.629	0.623
FLOW2	0.474	0.585	0.594	0.605	0.908	0.494	0.669	0.640
FLOW3	0.526	0.552	0.682	0.610	0.852	0.552	0.685	0.666
PEOU1	0.487	0.447	0.498	0.482	0.511	0.889	0.486	0.622
PEOU2	0.503	0.495	0.475	0.512	0.534	0.926	0.524	0.644
PEOU3	0.633	0.564	0.505	0.597	0.533	0.890	0.534	0.697
PU1	0.604	0.555	0.693	0.613	0.624	0.494	0.874	0.656
PU2	0.552	0.558	0.683	0.601	0.671	0.499	0.931	0.632
PU3	0.537	0.496	0.723	0.573	0.632	0.458	0.884	0.604
PU4	0.534	0.593	0.638	0.600	0.722	0.580	0.885	0.716
SMPUR1	0.614	0.561	0.569	0.716	0.640	0.695	0.605	0.909
SMPUR2	0.586	0.589	0.614	0.674	0.628	0.629	0.648	0.892
SMPUR3	0.606	0.638	0.650	0.694	0.665	0.618	0.703	0.871

*CON, concentration; ENJ, enjoyment; FEED, feedback; TIME, time distortion; FLOW, flow experience; PEOU, perceived ease of use; PU, perceived usefulness; SMPUR, social media purchase intention. The highlighted yellow values represent the factor loading value of every indicator exceeding the factor loading value of all the other constructs in the latent structure*.

The goodness of fit (GOF) for this research study was analyzed using the proposed model of Tenenhaus et al. (2005), to determine the quality of the proposed research model, which was calculated as followed:


$$\begin{array}{l} GOF=\sqrt{\bar{AVE}}\;x\;\sqrt{\bar{R^{2}}}=\;\sqrt{0.773\;x\;0.549}=0.651 \end{array}$$

According to the aforementioned result, the GOF is 0.651, which reaches the 0.349 cut-off criteria for a large impact size (Wetzels et al., 2009).

### Inner Model Result and Hypotheses Testing

**Figure 2:**
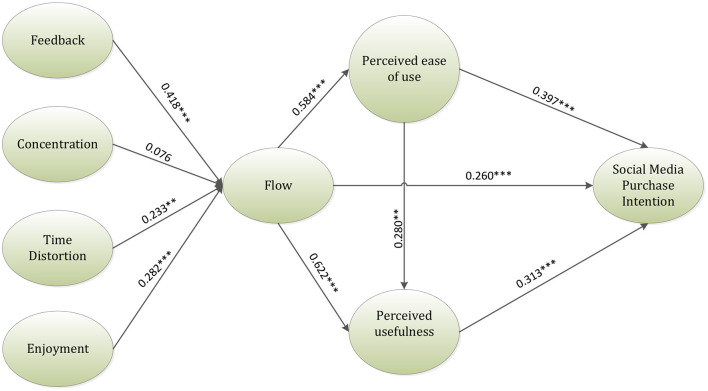
A framework of the inner model result. **p ≤ 0.01, ***p-value <0.001.

The hypothesis of this study was analyzed using the internal PLS model. The direction and strength of each latent and measured construct are determined by the values of path coefficients. Additionally, the value of the R square signifies the percentage of predictor variables, which demonstrates the predictive capacity of the model. The degree of each path coefficient was analyzed by using the bootstrapping approach. Data resampling method was used to re-extract the data, which was more accurate than the normal estimated value (Purvis et al., 2001). This research, hence, utilized this methodology to verify the relationship between the constructs. Table 3 and Figure 2 reveal the inner model results of the hypotheses. According to the results indicated by Table 3, all the hypotheses except hypothesis 2 were accepted. The variables in hypothesis 2 (β = 0.076, *p* = 0.018) were not in a significant relationship. The antecedents, such as feedback, enjoyment, and time distortion were found to be in a positive relationship with flow experience; however, the concentration did not have a significant effect on the flow. Furthermore, the flow was found to be in a significant relationship with social media purchase intention and TAM. Finally, TAM also had a positive significant relationship with social media purchase intention.

**Table 3:** Hypotheses results.

**Hypothesis**	**Path coefficient**	***T*-values**	***P*-values**	**Results**
H1: Feedback -> flow	0.418	4.890	0.000	Supported
H2: Concentration -> flow	−0.076	1.316	0.188	Not supported
H3: Time distortion -> flow	0.233	2.867	0.004	Supported
H4: Enjoyment -> flow	0.282	3.827	0.000	Supported
H5: Flow -> social media purchase intention	0.260	3.715	0.000	Supported
H6: Flow -> perceived ease of use	0.584	10.914	0.000	Supported
H7: Flow -> perceived usefulness	0.622	13.459	0.000	Supported
H8: Perceived ease of use -> social media purchase intention	0.397	6.546	0.000	Supported
H9: Perceived usefulness -> social media purchase intention	0.313	3.796	0.000	Supported
H10: Perceived ease of use -> perceived usefulness	0.208	3.352	0.001	Supported

## Discussion

Based on the integrated theoretical framework of antecedents of flow and TAM, this paper investigates the way flow experiences drive the intention to engage in SNSs shopping. Thus, the findings are noteworthy. According to the findings, all the antecedents were found to be in a significant relationship with the flow. The results of the hypothesis were somewhat like a study conducted by Barta et al. (2021). However, concentration was not found to be in a significant relationship with flow experience. This implies that enjoyment, time distortion, and feedback are more significant constructs in generating the optimal flow experience as compared to concentration.

Furthermore, the significant effect of flow on the SNSs for purchasing specifies that customers believe social media purchasing is an entertaining and enjoyable experience. This suggests that as the degrees of flow of customers rise, the intent for social media shopping is expected to be significantly optimistic. Moreover, the results are also in line with the previous studies (Gao and Bai, 2014; Ozkara et al., 2017; Chen et al., 2018; Rahman et al., 2020). Additionally, the outcomes show that flow boosts the usefulness and ease of use of social media shopping. The findings are somewhat similar to a recent study by Hyun et al. (2021). Their positive impacts likewise describe the total concentration in a buying activity and enjoyment of pleasurable attributes of the communication with shopping platforms on SNSs, for instance, unrelated stimuli are removed whenever social media users are having a complete engagement with SNSs. It is noted that compared to the ease of use, the usefulness of SNSs used for shopping is in a much more significant relationship with the flow, which indicates that the flow reduces adverse cognition for SNSs and improves the usefulness of SNSs used for shopping whenever users are experiencing flow.

In addition, this study discovers that usefulness and ease of use have a significant impact on social media purchase intention. Although analyzing these relations is not innovative; however, less information is available regarding the influence of these two constructs, when the antecedents and theory of flow are incorporated with the technology acceptance model to analyze the social media purchase conducts. Furthermore, this research study broadens the use and implementation of earlier theories to social media purchasing. Moreover, the outcome that indicates a significant impact of PU on the social media purchase intention is consistent with earlier researches (e.g., Shen, 2012; Yahia et al., 2018; Cutshall et al., 2020), and a positive association between PEOU and social media purchase intention is similar to a recent research (Hyun et al., 2021). The essential position of usefulness can suggest that customers consider that utilizing SNSs for purchasing is beneficial since it lets them explore products and buy at the same spot. This arrangement improves purchase efficiency. Though social media is deemed useful for shopping, once customers frequently make use of it, they get acquainted with it and do not think its ease of use is an essential component.

## Theoretical Implications

Although the senior market signifies an enormous, rapidly expanding segment, fewer investigations have been done on this segment following a theoretical model devised for determining the social media purchase intention. This research has several contributions. First, the findings have theoretical implications for the research related to flow. Essential variations were noted regarding the flow antecedents. This research incorporates antecedents of flow experience with flow theory and the TAM to analyze the social media purchase intention of the customers. Thus, this research study expands the theory of flow in the social media buying perspective and combines it with the TAM. The results explain that flow can boost optimistic assessments of usefulness and ease of use. It highlights that experiencing flow while using SNSs will filter out the unrelated stimuli, making social media purchases useful and easy. It additionally demonstrates that flow raises the probability of social media purchase intention. It increases the use of TAM in social media shopping and surfaces the path for identifying existing factors of customer social media purchase intention.

Furthermore, this research validates the important role of PU in the social media purchase intention of senior citizens. Previously, most of the senior citizens believed that the internet and use of technology were irrelevant to their daily lives, and hence, there were a large number of “voluntary non-users” in senior citizens (Frissen, 2005; Barta et al., 2021). Hence, future studies can help senior citizens by communicating the benefits of the internet and social media usage to their daily lives. Moreover, according to the results of this study, PEOU is a significant factor in the social media purchase intention of senior citizens, therefore, future studies can target to incorporate the necessary features, for instance, larger fonts, bright colors, clear audio, and video applications in designing a friendly user interface for senior citizens. The user-friendly interface can reduce anxiety and in turn generate a flow experience in senior citizens.

## Practical Implications

The constantly expanding volume of the senior market segment makes this research to be a significant contribution in focusing the utilization of social media by this population for buying, hence, producing highly valuable knowledge for manufacturers, wholesalers, vendors, and a huge number of senior customers. Additionally, this study has important practical contributions.

The outcomes provide vital evidence for companies, particularly, for their social media sales management. Great organization of content in online context supports to improve customer loyalty by improving purchase intention; hence, proper online content can assist customers to experience flow. The current research additionally offers numerous valuable intuitions into SNSs business operators and sellers. As demonstrated in the findings, the flow has a significant impact on the usefulness, PEOU, and social media purchase intention. These findings advise managers regarding two factors. First, flow is the basis for growing optimistic views of technology and its implementation concerning social media shopping. Second, PU and ease of use play as the key elements that boost social media purchase intention. From these significant results, marketers must believe this as a chance to merge technical expertise with flow attributes to build shopping features that generate complete experiences and improve opinions of social media shopping, which is simpler, more helpful, and attractive. Concerning the usefulness and ease of use, these two key components must be stressed if companies need to improve the social media shopping experience and efficiency of customers. Contrasting other conventional e-commerce platforms, SNSs for buying must be intended to lessen the effort, improve productivity, and boost a comfortable shopping experience.

Furthermore, practitioners are advised to consider the flow approach while designing the marketing strategies for each step of the purchasing process for senior citizens to reduce the anxiety involved in the purchase process. Customer frustration experienced because of any step of the purchase process can result in the flow experience disappearing (Herrando et al., 2018). Hence, to reduce the adverse impacts of other phases of the buying process, SNSs must implement strategies to make the customer informed regarding the likelihood of flow, provided the optimistic answers this state induces. Most of the time, customers in a state of flow are unaware of their experience. Hence, asking questions after their purchase can encourage the customer to remember the flow experience, which can enhance the constructive impacts of flow, and lessen the influence of adverse characteristics that might have arisen alongside the journey of the buyer, and finally boost the online reviews. Given that, online reviews are considered to be important in both online and offline purchases (Orús et al., 2019).

## Limitations

This study has limitations that must be noted considering future research. First, the research was conducted in just one country, so findings do not apply to other countries, and caution should be shown about understanding these findings because of this limitation. It is thus suggested that scholars perform similar research across different geographic areas all over the world to assess these results. This research was conducted in an emerging country, future studies can target developed economies. Furthermore, a cross-cultural comparison can be conducted between the findings from emerging and developed economies. Second, one would argue that flow can be viewed as a multi-dimensional construct while it is described as a single-dimensional construct in this research. Hence, there is a shortage of comprehensive knowledge of flow and its correlation with PU, ease of use, and purchase intention of SNSs. Upcoming research is supported to incorporate multi-dimensional flow constructs and analyze them. Finally, the research framework of this study is based on the flow antecedents and theory, and the TAM to describe the social media purchase intention of customers. It is considered that more theoretical models, such as self-determination theory, could be incorporated into future studies to enhance their utility in describing customer activities and marketing of social media shopping.

## Data Availability Statement

The raw data supporting the conclusions of this article will be made available by the authors, without undue reservation.

## Ethics Statement

Ethical review and approval was not required for the study on human participants in accordance with the local legislation and institutional requirements. Informed consent was obtained from all subjects involved in the study.

## Author Contributions

YX and AK: conceptualization. YW and AK: formal analysis. AK: investigation. YW and AK: methodology. YW and RZ: validation. YX, YW, AK, and RZ: writing–original draft and writing, reviewing, and editing. All the authors have read and agreed to the published version of the manuscript.

## Conflict of Interest

The authors declare that the research was conducted in the absence of any commercial or financial relationships that could be construed as a potential conflict of interest.

## Publisher's Note

All claims expressed in this article are solely those of the authors and do not necessarily represent those of their affiliated organizations, or those of the publisher, the editors and the reviewers. Any product that may be evaluated in this article, or claim that may be made by its manufacturer, is not guaranteed or endorsed by the publisher.
